# Supplementary material for: Content-rich biological network constructed by mining PubMed abstracts
Source: BMC Bioinformatics. 2004 Oct 8;5:147. doi: 10.1186/1471-2105-5-147 (PMC528731; doi:10.1186/1471-2105-5-147)
Supplement: Additional File 2 — The original results of the above study (non-essential files are deleted to keep the file size under the limit set by BMC bioinformatics). [file 1471-2105-5-147-S2.bz2 › chilibotAdditionalFile2/dip10/462ID10498628E10483/html/CCR9_CCL25.html]

 


 **CCR9** and **CCL25** 
  
Found 24 abstracts in PubMed, retrieved 10.  
 

 What does Google say? 
 PDF only 
| .edu only 

---

**Interactive relationship** (e.g. stimulation, inhibition, etc)

**Neutral relationship**- Role of the CC chemokine receptor 9  [ **CCR9** ]  TECK  [ **CCL25** ]  interaction in apoptosis.  Ref: 11997671 Apoptosis, 2002
- All  **CCR9**  thymocyte subsets migrated in response to  **CCL25** .  Ref: 11751956 J Immunol, 2002
- **CCR9**  mediates chemotaxis in response to  **CCL25**  thymus expressed chemokine  [ **CCL25** ]  and is selectively expressed on T cells in the thymus and small intestine.  Ref: 11884450 J Immunol, 2002
- Freshly isolated murine CD8alpha alpha CD3 IEL migrated in response to  **CCL25**  and expressed the  **CCL25**  receptor,  **CCR9** .  Ref: 12442331 Eur J Immunol, 2002
- **CCR9**  mediates chemotaxis of thymocytes in response to  **CCL25**  thymus expressed chemokine  [ **CCL25** ] , and its mRNA is selectively expressed in thymus and small intestine, the two known sites of T lymphopoiesis.  Ref: 11751956 J Immunol, 2002
- Characterization of  **CCR9**  expression and  **CCL25**  thymus expressed chemokine  [ **CCL25** ]  responsiveness during T cell development CD3 high CD69 thymocytes and gammadeltaTCR thymocytes preferentially respond to  **CCL25** .  Ref: 11751956 J Immunol, 2002

**Non-interactive relationship** (e.g. studied together, co-existance, homology, etc.)

- In contrast,  **CCR9** , whose ligand TECK  [ **CCL25** ]   **CCL25**  is predominantly restricted to the small intestine and thymus, is expressed by a fraction of IgA Ab secreting cells and almost all T cells in the small intestine, but by only a small percentage of plasma cells and plasmablasts in other sites.  Ref: 12671049 J Clin Invest, 2003
- Thus, oral antigens induce effector memory cells that express essential receptors for intestinal homing, namely the integrin alpha4beta7 and  **CCR9** , the receptor for the gut associated chemokine TECK  [ **CCL25** ]   **CCL25**  refs, 9.  Ref: 12840763 Nature, 2003
- Thus, these results demonstrate that the  **CCL25**   **CCR9**  pathway is essential for CP formation and the consequent appearance of IEL.  Ref: 12096027 Int Immunol, 2002
- Pivotal role of  **CCL25**  TECK  [ **CCL25** ]   **CCR9**  in the formation of gut cryptopatches and consequent appearance of intestinal intraepithelial T lymphocytes.  Ref: 12096027 Int Immunol, 2002
- These recently activated  **CCR9**  CD8alphabeta lymphocytes selectively localized to the small intestinal mucosa, and in vivo neutralization of the  **CCR9**  ligand,  **CCL25** , reduced the ability of these cells to populate the small intestinal epithelium.  Ref: 12393847 J Clin Invest, 2002
- Approximately one half of all gammadeltaTCR thymocytes and peripheral gammadeltaTCR T cells expressed  **CCR9**  on their surface, and these cells migrated in response to  **CCL25** .  Ref: 11751956 J Immunol, 2002
- Together these results demonstrate an important role for chemokines in the localization of T lymphocytes to the small intestinal mucosa and suggest that targeting  **CCL25**  and or  **CCR9**  may provide a means to selectively modulate small intestinal immune responses.  Ref: 12393847 J Clin Invest, 2002
